# The specificity of cluster training effects in sports: a systematic review and meta-analysis

**DOI:** 10.3389/fphys.2025.1722401

**Published:** 2026-01-19

**Authors:** Xinyi Zhao, Xudong Ya, Hao Zhou, Guowei Liu, Ziyao Liu, Jiaxin Luo, Yujia Liu, Yifeng Bu

**Affiliations:** 1 Institute of Physical Education, Jiangsu Normal University, Xuzhou, Jiangsu, China; 2 School of Vocational and Continuing Education, Ningxia Normal University, Guyuan, Ningxia, China; 3 Functional Nano & Soft Materials Laboratory of Soochow University, Suzhou, China

**Keywords:** cluster structure, meta-analysis, resistance training, strength and conditional training, training effect

## Abstract

**Objective:**

To systematically evaluate cluster structure training (CS) and traditional training (TS) in enhancing athletes’ motor abilities and explore sport-specific effects.

**Methods:**

Systematic searches of PubMed, Embase, Cochrane, and Web of Science (inception to March 2025). Quality was assessed using TESTEX, with meta-analyses calculating SMD (P < 0.05) and subgroup analyses by sport.

**Results:**

A total of 11 studies were included, showing that CS outperformed TS in improving athletes’ sprint ability (SMD = −0.32, 95% CI: [−0.56, −0.07], P = 0.012), explosive power (SMD = 0.39, 95% CI: [0.10, 0.68], P = 0.009) with significant differences. Subgroup analysis further revealed sport-specific effects: CS was more effective than TS in enhancing maximum strength (SMD = 0.30, 95% CI: [0.01, 0.59], P = 0.043), explosive power (SMD = 0.96, 95% CI: [0.26, 1.65], P = 0.007), and sprint ability (SMD = −0.65, 95% CI: [−1.15, −0.16], P = 0.009) in volleyball athletes, as well as peak power in soccer athletes (SMD = 0.68, 95% CI: [0.01, 1.36], P = 0.047).

**Conclusion:**

CS benefits volleyball and soccer, where explosive power is key. Coaches should tailor CS to sports’ energy demands and work-rest ratios.

**Clinical Trial Registration:**

https://www.crd.york.ac.uk/PROSPERO/home, identifier CRD420251015968.

## 1 Introduction

Traditional resistance training typically employs a fixed arrangement of sets and repetitions, where athletes complete a specified number of repetitions consecutively, followed by 1–5 min of inter-set rest configuration referred to as “traditional sets” in the literature (Tufano et al., 2017), training conducted using this approach is referred to as Traditional structure training (TS). To achieve different training objectives, elements such as exercise order, resistance load, number of sets, repetitions per set, rest intervals duration, and training frequency are considered and designed when formulating resistance training programs (Carroll et al., 2001). The core of this approach lies in designing the number of training sets and repetitions in accordance with specific training objectives such as muscle hypertrophy, muscle strength, explosive power, and muscular endurance within the framework of conventional continuous training set configurations.

Cluster structure training (CS) is a training modality in which traditional resistance training sets are divided into multiple clusters (i.e., exercise blocks) consisting of fewer repetitions, with brief rest intervals of 15–45 s inserted between consecutive clusters (Haff et al., 2008). This model reconstructs the training structure by segmenting individual sets and increasing the frequency of rest periods (Iglesias-Soler et al., 2014). Guided by the core concept of CS, several researchers have developed various configurations of cluster structures, with five representative forms: Intra-set Rest, Inter-repetition Rest, Rest Redistribution, Rest-Pause Method, and Equal Work-to-Rest Ratio (Tufano et al., 2017). However, some scholars argue that in the transition from TS to CS training, the modality involving adding rest intervals between exercise blocks should be termed CS, whereas the modality involving redistributing total time across exercise blocks should be referred to as Rest Redistribution (Jukic et al., 2020). Nevertheless, other researchers have opted not to distinguish Rest Redistribution from other cluster structures in their studies, instead examining the combined differences between these cluster structures and traditional structure. For the sake of facilitating the analysis of differences among research projects, this paper will also not differentiate between the two. Given that different set configurations can affect adaptive responses (Tufano et al., 2017), investigating the impacts of modifications to set configurations during resistance training is of great significance.

The differences between CS and TS are primarily reflected in the speed and power. During TS training, as the degree of fatigue increases, the work rate of single movements shows a gradual downward trend. In the high pull training of weightlifters, it has been found that using the CS model can effectively maintain the peak velocity and peak displacement of the barbell (Haff et al., 2003). Compared with TS, CS facilitates elimination of fatigue by providing more frequent rest periods, which helps maintain phosphocreatine storage and accelerate the clearance of metabolic products (Denton and Cronin, 2006; Girman et al., 2014; Oliver et al., 2015). Subjects also exhibit lower levels of central and peripheral nervous fatigue, specifically manifested as higher maximum voluntary contraction intensity, voluntary electromyographic activity, low-frequency fatigue index, single/double electric pulses, and the rate of force development and relaxation during single contractions (Río-Rodríguez et al., 2016). Meanwhile, CS can significantly reduce hemodynamic and cardiovascular stress, as indicated by lower heart rate and heart rate-blood pressure product (Río-Rodríguez et al., 2016). These advantages enable resistance training to maintain a relatively higher power output and effectively sustain movement speed. Therefore, CS is widely regarded as an effective training method for enhancing explosive power, especially in the later stages of continuous movements that place a high emphasis on power output (Wetmore et al., 2019).

Although existing studies have explored the effects of CS on muscle strength (Burd et al., 2010; Oliver et al., 2013), explosive power (Iglesias et al., 2010; Oliver et al., 2013), endurance (Farias et al., 2019; Dias et al., 2020), and muscle hypertrophy (Oliver et al., 2013; Iglesias-Soler et al., 2017), no direct evidence has confirmed which type of athlete is more sensitive to CS. From the perspective of event characteristics: In sports dominated by single explosive movements such as high jump, long jump, shot put, javelin throw, and weightlifting, athletes have lower requirements for long-term anti-fatigue ability, with training goals focusing more on improving the speed, power, and explosive performance of single movements. However, during training, fatigue induced by both central nervous and peripheral muscle factors significantly reduces motor dynamics efficiency (Zając et al., 2015; Enoka and Duchateau, 2016). The deceleration of the speed, strength and power output of the movements is likely to lead to the failure of repetitive actions (Sánchez-Medina and González-Badillo, 2011). CS can, to a certain extent, compensate for such declines in performance during explosive training by altering the training structure. In continuous combat events, team sports, or middle-distance sprints (such as wrestling, judo, soccer, 400 m), greater attention is paid to athletes’ ability to maintain high movement quality and power output under fatigued conditions during competitions. Based on this, it can be inferred that differences in energy metabolism characteristics and movement patterns among different sports may lead to significant differentiation in the applicability and effectiveness of CS.

This study aims to compare the differential effects of CS and TS on improvements in various sport-specific performance indicators through systematic review and meta-analysis, with aggregated analyses conducted on homogeneous or comparable test contents across studies and subgroup analyses performed stratified by sport. This research design provides substantial value to strength and conditioning practitioners and sports professionals by enhancing their accurate understanding of the adaptive characteristics of different training modalities within specific sporting contexts; it further facilitates the optimization of load distribution and cycle design in training programs to meet the unique requirements of different sports, while serving as an evidence-based foundation for developing personalized training plans that better address the distinct needs of athletes across various sporting disciplines.

## 2 Methods

This study protocol was registered in the PROSPERO database (Registration Number: CRD420251015968; 20 March 2025) and reported in accordance with the PRISMA guidelines (Vrabel, 2009). Based on the PRISMA guidelines, the research question was defined using the PICOS framework as follows: (1) Participants: Male or female athletes specializing in specific sports; (2) Intervention: Cluster structure training (CS); (3) Comparison: Traditional structure training (TS); (4) Outcome measures: Performance related parameters including maximal strength, sprint ability, explosive power, peak power output, and agility; (5) Study design: Prospective randomized controlled trials (RCTs).

### 2.1 Search strategy

A systematic search was conducted in four databases: PubMed, Embase, Cochrane Library, Web of Science. The search strategy combined MeSH terms and keywords, with the specific search formula as follows: (resistance exercise) OR (Training, Resistance) OR (Strength Training) OR (Weight-Lifting Strengthening Program) OR (Weight-Lifting Exercise Program) OR (Weight-Bearing Strengthening Program) OR (Weight-Bearing Exercise Program)AND (cluster set) OR (cluster training) OR (cluster) OR (cluster loading) OR (cluster-type) OR (rest-pause) OR (traditional set) OR (intra set) OR (inter rep) OR (work-to-rest ratio) OR (rest redistribution) OR (rest-loading) AND (Athlete) OR (Professional Athletes) OR (Athlete, Professional) OR (Athletes, Professional) OR (Elite Athletes) OR (Athlete, Elite) OR (College Athletes) OR (Athlete, College) OR (sportswoman) OR (sportsman) OR (player) OR (players). The literature search was not restricted by language, with eligible study types confined to peer-reviewed journal articles. Gray literature, meeting minutes, and records from trial registries were explicitly excluded. The complete search strategy is detailed in the supplementary materials (Supplementary Material S1). Two independent researchers conducted systematic searches for potential studies published on or before 18 March 2025. Any discrepancies arising during the search and screening process were resolved through consultation with a third reviewer. Additionally, the reference lists of all included studies were hand-searched to identify any additional eligible studies that might have been missed in the initial database searches. The specific search terms for each database are provided in the Supplementary Material S1.

### 2.2 Eligibility criteria

Studies were included in this review if they met the following criteria:

(1) Training intervention group that utilized a cluster structure configuration (i.e., intra-set rest, inter-repetition rest, rest redistribution and/or rest-pause models) as defined by Tufano et al. (2017); (2) Specified the sport discipline of participants; (3) Reported data as mean ± standard deviation (SD) directly or allowed data extraction from figures; (4) Included participants of all age groups; (5) Recruited participants without any health conditions or injuries; (6) The training volume and intensity of the experimental (CS) group and the control (TS) group were required to satisfy *a priori* matching criteria: the discrepancy in total training load between the two groups must not exceed 10% (Buresh and Berg, 2014); (7) Intervention duration should be no less than 2 weeks.

### 2.3 Literature exclusion criteria

Studies were excluded if they met any of the following criteria:

(1) Acute intervention studies; (2) Non-human studies; (3) Dissertation/thesis publications; (4) Studies implementing CS alone without comparison to TS.

### 2.4 Literature quality assessment

The quality assessment was conducted by two independent researchers using the Training Study Evaluation and Reporting Tool (TESTEX Scale) (Smart et al., 2015), which is specifically designed for exercise training research and provides comprehensive assessment of both study quality (5 points) and reporting standards (10 points). The study quality assessment criteria include: specified eligibility criteria (1 point); specified randomization (1 point); allocation concealment (1 point); baseline homogeneity (1 point); and assessor blinding (1 point). Reporting evaluation encompassed: outcome measurement documentation including participants adherence, adverse events, and attendance records (3 points); intention-to-treat analysis (1 point); between-group statistical comparisons for primary and secondary outcomes (2 points); selective outcome reporting (1 point); activity monitoring (1 point); intensity control measures (1 point); exercise volume and energy expenditure measurable (1 point). Disagreements were resolved through consensus or by a third assessor. The maximum achievable score was 15 points, with quality classifications based on quartile distribution: <4 points (poor quality), 4-7 points (moderate quality), 8–11 points (good quality), and >11 points (excellent quality) (Davies et al., 2021).

### 2.5 Data extraction

For all included studies, the following data were extracted: (1) Study characteristics (author, year, sample size, intervention duration, intervention modality) and participant demographics (age, gender, sport); (2) The mean difference (change) and SD between baseline and post-intervention measurements for both CS and TS were calculated using two formulas, specifically:

Mean change=Mean baseline−Mean post

$$\text{SD change}=\sqrt{{\text{SD}\;\text{baseline}}^{2}+{\text{SD}\;\text{post}}^{2}-\left(2\times R\times \text{SD baseline}\times \text{SD post}\right)}$$

The formula was chosen because calculating the difference between baseline and post-test data in individual studies allows evaluation of intervention efficacy (Higgins et al., 2019). R represents the correlation coefficient between the baseline measurement before the intervention and the post measurement value after the intervention (Rosenthal, 1991). The change in standard deviations was calculated in accordance the guidelines provided in the *Cochrane Handbook for Systematic Reviews of Interventions* (Chandler et al., 2019) and a conservative correlation coefficient of 0.5 was adopted to ensure the inclusion of the maximum number of studies in the meta-analysis (Elbourne et al., 2002; Zhang et al., 2025). Extracted data included: (3) Training protocols (rest intervals, repetitions, sets, CS configurations, exercise selection, and intensity for both CS and TS); (4) Outcome measures: maximal strength, sprint ability, explosive power, peak power output, and agility; (5) When necessary, raw data presented in figures were extracted using GetData Graph Digitizer software.

### 2.6 Data analysis

The first step was to calculate the outcome changes from baseline to post-intervention in the experimental and control groups, followed by subgroup analysis based on participants’ sport disciplines. Meta-analysis was performed using STATA 18.0 (The specific code is available in Supplementary Material S2) Given the measurement heterogeneity of indicators (e.g., sprint ability, explosive power, lower-limb explosiveness, peak power, and maximal strength of upper/lower limbs), the effect size (ES) was calculated using the standardized mean difference (SMD) with 95% confidence intervals (CI) were used for continuous data and set the significance level to p < 0.05. ES of 0.2 was considered a small effect, 0.5 a moderate effect and 0.8 a large effect (Hopkins et al., 2009). A positive ES indicates that the intervention effect favored CS, whereas a negative ES favored TS. This pattern is reversed for sprinting and agility tasks, where performance is optimized by shorter completion times. Heterogeneity was assessed *via* Cochran’s Q test and Higgins’ I^2^ statistic: P < 0.10 or I^2^ > 50% indicated substantial heterogeneity, warranting a random-effects model; otherwise, a fixed-effects model was applied (Higgins et al., 2003). Subgroup analysis was conducted to explore heterogeneity sources when applicable. Significance was determined *via* the Z test, with pooled results showing statistical significance at P < 0.05. Sensitivity analysis was performed *via* leave-one-out cross-validation (i.e., removing one study per iteration) to evaluate individual studies’ impact on pooled estimates. For publication bias and small-sample bias assessment, Egger’s test and Begg’s rank correlation test were used: P > 0.05 suggested no significant bias (Schneck, 2017), while P ≤ 0.05 indicated potential bias, which was corrected *via* nonparametric trim-and-fill methods if present.

## 3 Results

### 3.1 Study selection

**FIGURE 1:**
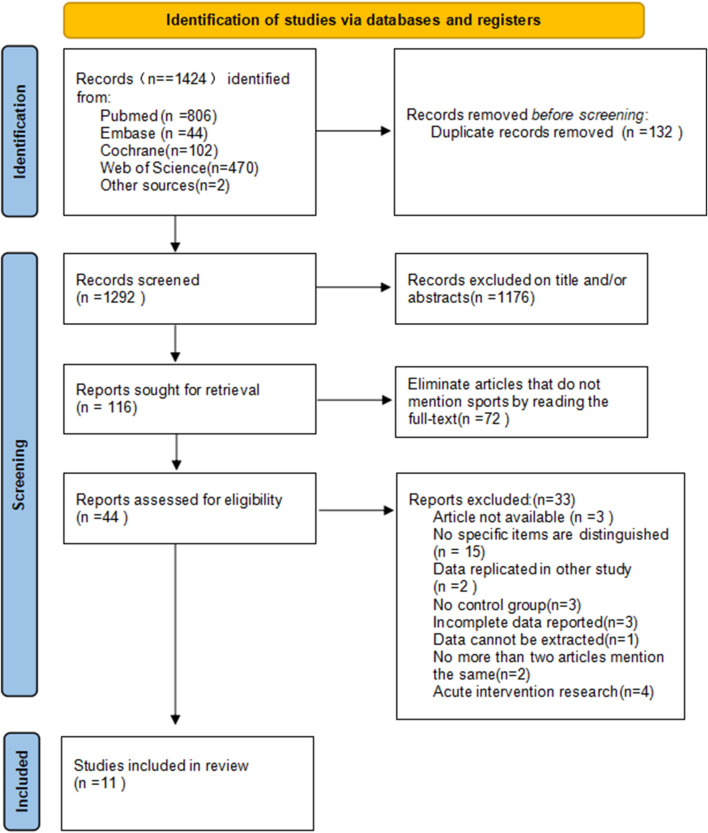
PRISMA flow diagram of the study selection process.

Figure 1 illustrates the literature search and screening process. A total of 1,424 potential studies were identified from electronic databases. After importing into NoteExpress software, 132 duplicate studies were removed. Following screening by titles and abstracts, 44 reports remained. Full-text review excluded 33 reports for the following reasons: inaccessible articles (n = 3), failure to specify sport disciplines (n = 15), duplicate data from other studies (n = 2), absence of control groups (n = 3), incomplete data reporting (n = 3), unextractable data (n = 1), insufficient studies for meta-analysis (i.e., under 2 articles on the same outcome; n = 2), and acute intervention designs (n = 4). Eleven studies were ultimately included (Hansen et al., 2011; Zarezadehmehrizi et al., 2013; Arazi et al., 2017; Cin et al., 2021; Yilmaz et al., 2021; Ersöz et al., 2022; Chen and Gao, 2023; Harris et al., 2024; Rong and Xiu, 2024; Zhu et al., 2024; Öztürk et al., 2025).

### 3.2 The basic characteristics of the included literature

The RCT studies were published between 2011 and 2025, with a sample size of 11–32 participants per group. Participants were aged 14–32 years, predominantly male. All participants were from six different sports disciplines. Specifically, three studies were included for volleyball, with a total of 64 participants; four studies for soccer, totaling 87 participants; one study for rugby, including 18 participants; one study for table tennis, with 32 participants; one study for judo, involving 22 participants; and one study for badminton, including 16 participants. The training frequency ranged from 2 to 4 sessions per week, and the median training duration was 8 weeks (range: 4–12 weeks). Detailed participant characteristics, specific intervention methods, loads, and other relevant information are presented in Table 1.

**TABLE 1:** Characteristics of included studies.

Study	Sport	Group	Age (years)	Height (cm)	Body weight (kg)	Sex (man/female)	Intervention characteristics								Outcome measure
							Frequency (times/week)	Stage (week)	Training content	Intensity (%1RM)	Set	Repetition	Rest between set(S)	CS rest interval (S)	
Arazi et al. (2017)	Volleyball	CS	18.2 ± 2.4	161 ± 6	54.5 ± 6.6	0/10	3	1–4	BS, BP, DL, MP	60–80	3	1 × 10, 2 × 5, 1 × 4 + 1 × 6	90–120	20–30	1RM (BS, BP, MP, DL), CVJ, peak power output (CVJ), 4 × 9mshuttle run, 20 m sprint
								5–8	SJ, EBP, DL, PC	30–90		1 × 6, 2 × 3, 3 × 5 × 2, 3 × 3 × 2, 3 × 1 × 2	120–180	10–30	
		TS	18.7 ± 1.5	166 ± 5	56.5 ± 9.0	0/10		1–4	BS, BP, DL, MP	60–80		8/10	120	—	
								5–8	SJ, EBP, DL, PC	30–90		4/6/8, 2× (1 × 6), 2× (1 × 4), 2× (1 × 3)	180	—	
Cin et al. (2021)	Volleyball	CS	27.1 ± 1.60	195.7 ± 1.49	94.14 ± 1.65	14/0	3	1–6	BP, MP, BS, DL, PO	85	3	2 × 3	80	20	1RM (BS, BP, PO, DL) CVJ, T-test, 10–20 m sprint
		TS	27.4 ± 1.37	194.4 ± 1.89	93.6 ± 1.25	14/0						6	120	—	
Rong and Xiu (2024)	Volleyball	CS-COX	18.3 ± 0.4	182.6 ± 7.5	78.5 ± 6.2	8/0	2	1–6	BS, LP, SJ, LJ	65	4	2 × 5/6/7/8	60	30	1RM (LP, BS) peak power output (Wingate)
		TS-COX	18.5 ± 0.4	183.1 ± 6.4	77.4 ± 5.1	8/0						10/12/14/16	90	—	
Ersöz et al. (2022)	Soccer	AEL-CS	18.78 ± 0.83	166.73 ± 8.61	69.59 ± 6.03	16/0	2	1–4	BS, HP	Eccentric 80%/Concentric 50%	3	2 × 4	120	20	1RM (HP, BS), 10–20–30 m sprint, Illinois test
		AEL-TS				16/0						8	180	—	
Öztürket al. (2025)	Soccer	CS	18.12 ± 0.35	174 ± 2	73.50 ± 3.62	8/0		1–8	CVJ, BJ, TJ, HJ, DJ	Body weight	8–10	2–3	—	10	CVJ, 10–20–30 m sprint, Zigzag test
		TS	18.50 ± 0.53	175 ± 4	72.25 ± 3.32	8/0					2–3	8–10	70/90	—	
Yilmazet al. (2021)	Soccer	CS	14.88 ± 0.92	173.2 ± 8.9	57.38 ± 7.79	9/0	2	1	SLJ, SJ, AF, DLH	Body weight	10	1 × 2	—	10	CVJ, SJ, SLJ, 10 m–20 m sprint, Zigzag test
								2–3	SLJE, SJ, DLH-LCH-FCH			1 × 2	—	10	
								4–5	SLJ, SJ, AF, DLH, LCH, SSJ			1 × 2	—	10	
								6	SLJ, SJ, DLH, LCH, FCH			1 × 2	—	10	
		TS	15.25 ± 0.70	171.2 ± 5.9	54.01 ± 8.33	8/0		1	SLJ, SJ, AF, DLH		2	10	90	—	
								2–3	SLJE, SJ, DLH-LCH-FCH						
								4–5	SLJ, SJ, AF, DLH, LCH, SSJ						
								6	SLJ, SJ, DLH, LCH, FCH						
Zarezadehmehriziet al. (2013)	Soccer	CS	24.68 ± 3.13	176 ± 0.41	71.68 ± 6.85	11/0	3/4	1–4	BS, LE, LC, HR, FC, BC, BP, IBP, LPD, BTNPWB, OPWB	60–80	3	9	60–90	—	1RM BS, peak power output (BS)
								5–7	BS, LG, LC, FC, BP	85	3	1 × 5	120	10–30	
								8–10	BS, SJ, BPT	30–80	5	1 × 5	120	10–30	
						11/0		1–4	BS, LE, LC, HR, FC, BC, BP, IBP, LPD, BTNPWB, OPWB	60–80	3	9	60–90	—	
		TS						5–7	BS, LG, LC, FC, BP	85	3	5	180	—	
								8–10	BS, SJ, BPT	30–80	5	5	180	—	
Hansenet al. (2011)	Rugby	CS	27.8 ± 4.5	185 ± 10	99.7 ± 10.5	9/0	2	1–2	FS, CP	80–90	5	1 × 6, 2 × 3	120–180	0–30	1RM BS, peak power output (0, 20, 40, 60 kg SJ)
								3–4	BS, CP	80–95		1 × 5, 1 × 2 + 1 × 3	120–180	0–30	
								5–6	BOXS, PC	20–95		1 × 6, 3 × 1	120–180	0–10	
								7–8	BS, SJ, PC			1 × 3/4/5, 2 × 2, 3 × 1	120–180	0–20	
		TS	25.7 ± 4.5	193 ± 10	107.3 ± 6.7	9/0		1–2	FS, CP	80–90		4/6/8	180	—	
								3–4	BS, CP	80–95		3/5/7	180	—	
								5–6	BOXS, PC	20–95		3/4/5/6	180	—	
								7–8	BS, SJ, PC			3/4/5	180	—	
Zhu et al. (2024)	Table tennis	CS-PT	19.4 ± 1.2	179.4 ± 4.1	77.8 ± 5.3	8/0	3	1–8	MBCP, MBS, STC, LMBD	5% Body weight	3/4	2 × 5/6	60	30	MBT, 1RM BP, peak power output (Wingate)
		TS-PT	19.7 ± 1.4	180.8 ± 5.5	79.2 ± 5.8	8/0						10/12	90		
		CS-RT	19.5 ± 1.8	179.4 ± 4.6	78.8 ± 4.2	8/0			BP, SP, DFR, CPS			2 × 5/6	60	30	
		TS-RT	19.6 ± 1.1	178.8 ± 4.5	76.2 ± 4.8	8/0						10/12	90		
Harriset al. (2024)	Judo	CS	17.1 ± 0.8	168.6 ± 8.3	61.1 ± 9.0	6/5	3	1–4	PC, BOXS, DL, SS, SLRD, PJ, PBR, BP, SABOR, LPP, WPU	65–85	4	2 × 3/5	—	—	CVJ, 1RM (BS, BP)
								5–8	BS, DL, SS, SLRD, IBP, PBR, SSP, WPU	80–85	3/4	2 × 3/4	—	45	
								8–12	MS, HT, BAT, BT, JS, HHP, BS, CP		3	2 × 3	—	45	
		TS	17.8 ± 1.4	171.8 ± 5.9	65.6 ± 6.6	7/4		1–4	PC, BOXS, DL, SS, SLRD, PJ, PBR, BP, SABOR, LPP, WPU	65–85	4	6/10	—	—	
								5–8	BS, DL, SS, SLRD, IBP, PBR, SSP, WPU	80–85	3/4	5–6/8	—	—	
								8–12	MS, HT, BAT, BT, JS, HHP, BS, CP		3	6	—	—	
Chen and Gao (2023)	Badminton	CS	15.13 ± 1.356	171.63 ± 5.290	60.50 ± 7.764	8/0	2	1–6	BS	80	4	2 × 3	90	30	1RM BS, 30 m sprint, CVJ
		TS	15.25 ± 1.282	173.38 ± 9.257	60.88 ± 10.494	8/0						6	120	—	

Abbreviations: AEL, accentuated eccentric load; AF, angle flip; BAT, bridge and toss (dead ball) (osaekomi-waza); BC, barbell curl; BJ, broad jump; BP, bench press; BPT, bench press throw; BOXS, box squat; BS, back squat; BT, bench throws (smith machine); BTNPWB, behind the neck press; COX, resistance and plyometric training (complex); CP, clean pull; CVJ, countermovement vertical jump; DFR, dumbbell front rise; DL, deadlift; DLH, double leg hop; DJ, drop jump; EBP, explosive bench press; FC, french curl; FCH, front cone hop; FS, front squat; HHP, hang high pull; HJ, hurdle jump; HT, hip thrust; HR, heel raise; IBP, incline bench press; JS, jump shrug; LC, leg curl; LCH, lateral cone hop; LE, leg extension; LG, lunge; LJ, lunge jump; LMBD, lying medicine ball drop; LP, leg press; LPD, lat pull down; LPP, landmine push press; MBCP, medicine ball chest pass; MBS, medicine ball slams; MBT, medicine ball throw MS, midthigh snatch; PT, plyometric training; RT, resistance training; MP, military press; OPWB, overhead press with barbell; PBR, Prone bench (seal) row; PC, power clean; PJ, push jerk; PO, pull over; SJ, squat jump; SLJ, standing long jump; SSJ, split squat jump; SABOR, single-arm bent-over row; STC, seated throw circuit; SP, shoulder press; CPS, cable pulldowns; SS, split squat; SSP, seated shoulder press; TJ, tuck jump; WPU, weighted pull-u.

### 3.3 Quality evaluation of included literature

A total of 11 studies were included in this review, and their methodological quality was evaluated across 12 dimensions (Table 2), with quality scores ranging from 9 to 12 points and a median of 11 out of 15 points. In terms of literature quality, methodological limitations were widespread across core domains: only one of 11 studies reported randomization method, indicating inadequate performance in this aspect; allocation concealment was implemented in three out of 11 studies, leaving most studies at risk of selection bias; none of the included studies applied the intention-to-treat (ITT) principle, which may introduce potential attrition bias, and no study conducted assessor blinding, a finding likely attributed to the practical challenges associated with blinding procedures in exercise training research. Regarding reporting quality, five out of 11 studies achieved an outcome completion rate of ≥85%, and nine studies implemented dynamic adjustments to exercise intensity throughout the intervention period. Overall, while the reporting quality was generally robust, the core literature quality domains (randomization, allocation concealment, blinding, and ITT) exhibited pervasive limitations. This emphasizes the necessity of enhancing methodological rigor in the design of future exercise training studies.

**TABLE 2:** Quality evaluation results of included literature.

Study criterion	Arazi et al., 2017	Cin et al., 2021	Rong and Xiu, 2024	Ersöz et al., 2022	Öztürket al., 2025	Yilmazet al., 2021	Zarezadehet al., 2013	Hansenet al., 2011	Zhu et al., 2024	Harris et al., 2024	Chen and Gao, 2023
Eligibility criteria specified (1 point)	1	1	1	1	1	1	1	1	1	1	1
Randomization specified (1 point)	0	0	0	0	0	0	0	0	0	0	1
Allocation concealment (1 point)	0	1	0	1	0	0	0	1	0	0	1
Groups similar at baseline (1 point)	1	1	1	1	1	1	1	1	1	1	1
Blinding of assessor (1 point)	1	1	1	1	1	1	1	1	1	1	1
Outcome measures assessed in 85% of patients (3 point)	2	1	2	1	2	1	1	1	2	1	1
Intention-to-treat analysis (1 point)	0	0	0	0	0	0	0	0	0	0	0
Between-group statistical comparisons reported (2 point)	2	2	2	2	2	2	2	2	2	2	2
Point measures and measures of variability for all reported outcome measures (1 point)	1	1	1	1	1	1	1	1	1	1	1
Activity monitoring in control groups (1 point)	1	1	1	1	1	1	1	1	1	1	0
Relative exercise intensity remained constant (1 point)	1	0	1	1	1	1	0	1	1	1	0
Exercise volume and energy expenditure (1 point)	1	1	1	1	1	1	1	1	1	1	1
Total points	11	10	11	11	12	10	9	11	11	10	11
Quality	Good	Good	Good	Good	Excellent	Good	Good	Good	Good	Good	Good

### 3.4 Meta-analyses and subgroup analyses

#### 3.4.1 Maximal strength

**FIGURE 2:**
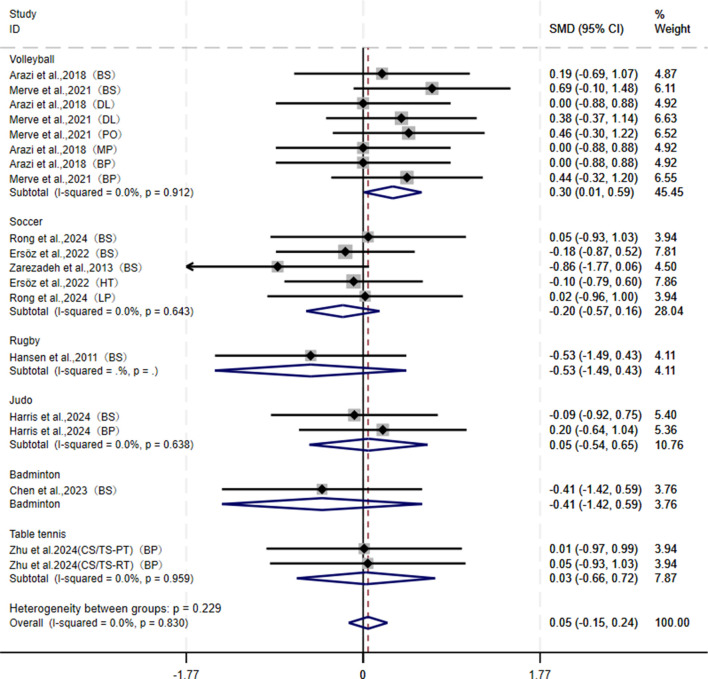
Forest plot of meta-analysis comparing maximal force between CS and TS in different sports. The dotted line represents the mean treatment effect. The diamond represents the overall treatment effect and 95% CI. BP, bench press; BS, back squat; DL, deadlift; HT, hip thrust; LP, leg press; MP, military press; PO, pull over.

In terms of maximal strength outcomes, this paper pooled 17 studies from nine articles involving back squat (BS), deadlift (DL), hip thrust (HT), leg press (LP), bench press (BP), military press (MP), and pull over (PO), which were further combined into upper-body and lower-body maximal strength indicators. Results showed that there was no significant overall difference in maximal strength improvement between CS and TS (I^2^ = 0.0%, SMD = 0.05, 95% CI: [−0.15, 0.24], P = 0.622). However, subgroup analysis indicated that CS was more effective than TS in improving maximal strength among volleyball athletes (I^2^ = 0.0%, SMD = 0.30, 95% CI: [0.01, 0.59], P = 0.043), with statistical significance. For athletes in soccer (SMD = −0.20, 95% CI: [−0.57, 0.16]), rugby (SMD = −0.53, 95% CI: [−1.49, 0.43]), table tennis (SMD = 0.03, 95% CI: [−0.66, 0.72]), judo (SMD = 0.05, 95% CI: [−0.54, 0.65]), and badminton (SMD = −0.41, 95% CI: [−1.42, 0.59]), no significant differences were observed between the two groups (Figure 2). Sensitivity analysis and publication bias tests (Table 3) demonstrated the robustness of the pooled results: Begg’s test (P = 0.552) and Egger’s test (P = 0.116).

**TABLE 3:** Results of Egger’s test and Begg’s publication bias test.

Outcome measure	Begg’s test	Egger’s test
Maximal force	0.552	0.116
Upper-body maximal strength	0.293	0.010
Lower-body maximal strength	0.732	0.302
Explosive power	0.032	0.214
Lower-body explosive strength	0.174	0.238
Peak power output	0.754	0.735
Sprinting ability	0.837	0.946
Agility and speed	0.221	0.066

**FIGURE 3:**
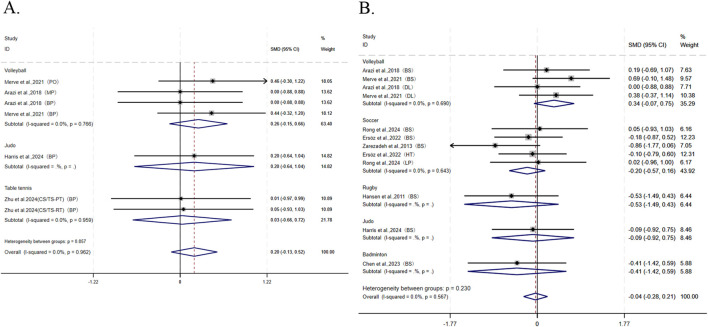
Forest plot of meta-analysis comparing maximum strength of upper and lower limbs between CS and TS in different sports. **(A)** Upper limbs. **(B)** Lower limbs. The dotted line represents the mean treatment effect. The diamond represents the overall treatment effect and 95% CI. BS, back squat; DL, deadlift; HT, hip thrust; LP, leg press.

In the eight articles reporting maximal strength, four articles reported seven studies on upper-body maximal strength. The results showed no significant difference in the improvement of upper-body maximal strength between CS and TS (I^2^ = 0.0%, SMD = 0.20, 95% CI: [−0.13, 0.52], P = 0.231) (Figure 3). Sensitivity analysis indicated the stability of the pooled results. Publication bias tests (Table 3) for upper-body maximal strength showed Begg’s test (P = 0.293) and Egger’s test (P = 0.01). This finding indicates a potential risk of publication bias. Consequently, a nonparametric trim-and-fill analysis was conducted, and the adjusted funnel plot exhibited symmetry. The original ES was 0.163 (95% CI: [−0.129 to 0.456]), while the post-adjustment ES was 0.186 (95% CI: [−0.090 to 0.463]). The minimal variation in ES, combined with the consistent crossing of the null effect line by the 95% CI, confirms a low level of bias and the absence of statistically significant publication bias (see Supplementary Material S3 for the funnel plot). Among the nine articles reporting maximal strength, eight articles reported 12 studies on lower-body maximal strength. The results showed no significant difference in the improvement of lower-body maximal strength between CS and TS (I^2^ = 0.0%, SMD = −0.04, 95% CI: [−0.28, 0.21], P = 0.777) (Figure 3). Sensitivity analysis and publication bias tests (Table 3) demonstrated the robustness of the pooled results: Begg’s test (P = 0.732) and Egger’s test (P = 0.302).

#### 3.4.2 Explosive power

**FIGURE 4:**
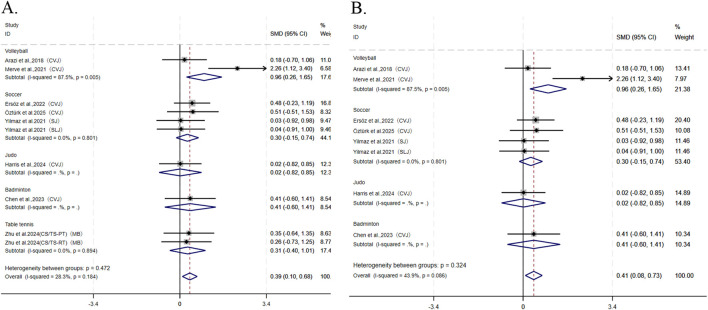
Forest plot of meta-analysis comparing explosive power and lower limb explosive power between CS and TS in different sports. **(A)** explosive power. **(B)** lower limb explosive power. The dotted line represents the mean treatment effect. The diamond represents the overall treatment effect and 95% CI. CVJ, countermovement vertical jump; SJ, squat jump; SLJ, standing long jump.

For the explosive strength outcomes, this study pooled 10 studies from eight articles involving countermovement vertical jump (CVJ), squat jump (SJ), standing long jump (SLJ), and MBT (medicine ball throw), which were further categorized as lower-body explosive strength indicators. Given that only two studies from a single article (Zhu et al., 2024) addressed upper-body explosive strength, no pooling was conducted for this subgroup. Overall, CS demonstrated significantly better enhancement of explosive strength compared to TS (I^2^ = 28.3%, SMD = 0.39, 95% CI: [0.39, 0.68], P = 0.009). Subgroup analysis revealed that CS was more effective than TS in improving explosive strength among volleyball athletes (I^2^ = 87.5%, SMD = 0.96, 95% CI: [0.26, 1.65], P = 0.007), whereas no significant differences were observed between the two groups for athletes in soccer (SMD = 0.30, 95% CI: [−0.15, 0.74]), table tennis (SMD = 0.31, 95% CI: [−0.40, 1.01]), judo (SMD = 0.02, 95% CI: [−0.82, 0.85]), and badminton (SMD = 0.41, 95% CI: [−0.60, 1.41]) (Figure 4). Sensitivity analysis confirmed the robustness of the pooled results. Publication bias tests (Table 3) for explosive strength showed Begg’s test (P = 0.032) and Egger’s test (P = 0.214), indicating a potential risk of publication bias. Consequently, a nonparametric trim-and-fill analysis was conducted, and the adjusted funnel plot exhibited symmetry. The original ES was 0.418 (95% CI: 0.145–0.691), whereas the post-adjustment ES was 0.583 (95% CI: 0.344–0.823). The minimal variation in ES confirms a low risk of bias and the absence of statistically significant publication bias (see Supplementary Material S4 for the funnel plot).

Among the eight articles reporting explosive strength, seven articles contributed seven studies on lower-body explosive strength. Results showed that CS outperformed TS in enhancing lower-body explosive strength (I^2^ = 43.9%, SMD = 0.41, 95% CI: [0.08, 0.73], P = 0.013). Subgroup analysis further demonstrated that CS was more effective than TS in improving lower-body explosive strength specifically among volleyball athletes (*I* ^2^ = 87.5%, SMD = 0.96, 95% CI: [0.26, 1.65], P = 0.007) (Figure 4). Both sensitivity analysis and publication bias tests (Table 3) supported the robustness of the pooled results: Begg’s test (P = 0.174) and Egger’s test (P = 0.238).

#### 3.4.3 Peak power output

**FIGURE 5:**
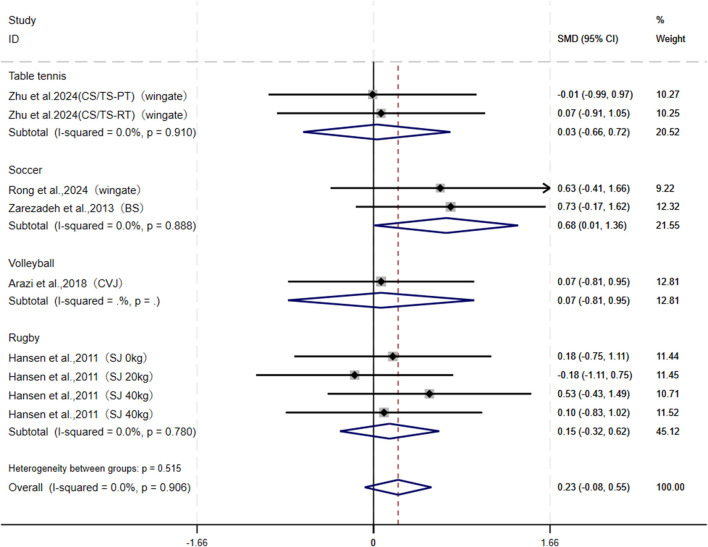
Forest plot of meta-analysis comparing peak power output between CS and TS in different sports. The dotted line represents the mean treatment effect. The diamond represents the overall treatment effect and 95% CI. Wingate, Wingate Power Bike; BS, back squat; CVJ, countermovement vertical jump; SJ, squat jump.

For the peak power output outcomes, this study pooled nine studies from five articles, which employed measurements including Wingate, CVJ, SJ, and BS. While combining different testing methods may be a source of heterogeneity, the statistical results indicate that the low level of heterogeneity is insufficient to cause substantial interference; this approach to pooling can expand the sample size and enhance the statistical power of the analysis. Notably, Zhu et al. (2024) reported two results from different structural resistance and plyometric training groups, while Hansen et al. (2011) provided four results of peak power output during squat jumps under 0, 20, 40, and 60 kg loads. Overall, no significant difference was observed between CS and TS in enhancing peak power output (I^2^ = 0.0%, SMD = 0.23, 95% CI: [−0.08, 0.55], P = 0.149). However, subgroup analysis revealed that CS was more effective than TS in improving peak power among soccer athletes (SMD = 0.68, 95% CI: [0.01, 1.36], P = 0.047), whereas no significant differences were found between the two groups for athletes in volleyball (SMD = 0.07, 95% CI: [−0.81, 0.95]), rugby (SMD = 0.15, 95% CI: [−0.32, 0.62]), and table tennis (SMD = 0.03, 95% CI: [−0.66, 0.72]) (Figure 5). Both sensitivity analysis and publication bias tests (Table 3) demonstrated the robustness of the pooled results: Begg’s test (P = 0.754) and Egger’s test (P = 0.735).

#### 3.4.4 Sprinting ability

**FIGURE 6:**
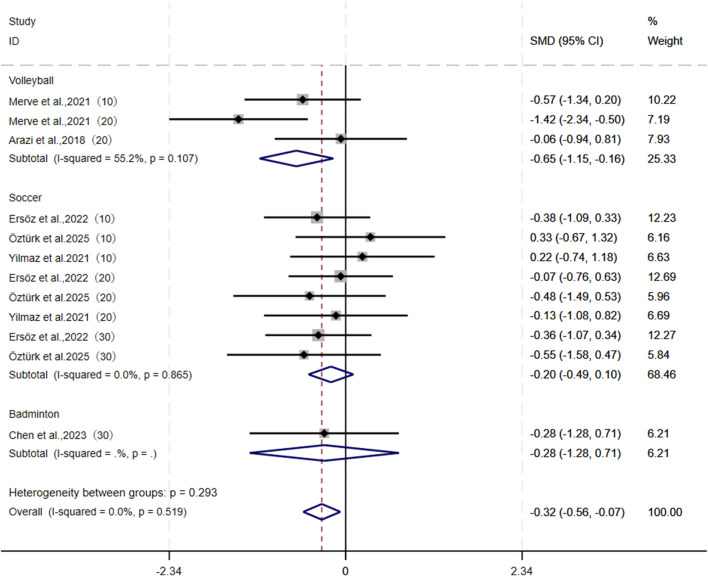
Forest plot of meta-analysis comparing sprinting ability between CS and TS in different sports. The dotted line represents the mean treatment effect. The diamond represents the overall treatment effect and 95% CI. 10:10 m sprint, 20:20 m sprint, 30:30 m sprint. A negative ES indicates that the intervention effect favored CS, whereas a positive ES favored TS.

For sprint performance outcomes, this study pooled 12 studies from six articles, all of which measured 10-20-30 m sprint times. A negative SMD was detected and showed that CS was significantly more effective than TS in enhancing sprint performance (I^2^ = 0.0%, SMD = −0.32, 95% CI: [−0.56, −0.07], P = 0.012). Subgroup analysis further revealed that CS outperformed TS specifically with a negative ES in improving sprint ability among volleyball athletes (SMD = −0.65, 95% CI: [−1.15, −0.16], P = 0.009). However, no significant differences were observed between the two groups for soccer players (SMD = −0.20, 95% CI: [−0.49, 0.10]) and badminton players (SMD = −0.28, 95% CI: [−1.28, 0.71]) (Figure 6). Both sensitivity analysis and publication bias tests (Table 3) confirmed the robustness of the pooled results: Begg’s test (P = 0.837) and Egger’s test (P = 0.946).

#### 3.4.5 Agility and speed

**FIGURE 7:**
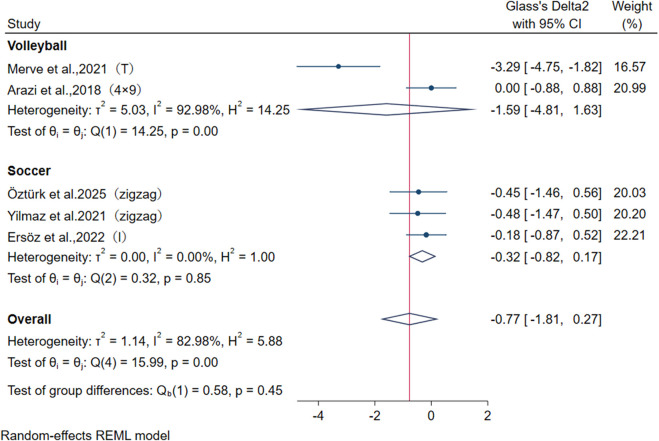
Forest plot of meta-analysis comparing agility and speed between CS and TS in different sports. The dotted line represents the mean treatment effect. The diamond represents the overall treatment effect and 95% CI. Zigzag, zigzag agility test; I, Illinois agility test; T, T agility test; 4 × 9:4 × 9 shuttle run. A negative ES indicates that the intervention effect favored CS, whereas a positive ES favored TS.

Regarding agility outcomes, this study pooled five studies from five articles, which employed various tests including the zigzag test, T-test, Illinois test, and 4 × 9 m shuttle run. Specifically, Arazi et al. (2017) used the 4 × 9 m shuttle run, Cin et al. (2021) applied the T-test, Ersöz et al. utilized the Illinois test, and both Öztürk et al. (2025) and Yilmaz et al. (2021) adopted the zigzag test. Overall, no significant difference was found between CS and TS in improving agility (I^2^ = 75.0%, SMD = −0.73, 95% CI: [−1.59, 0.13], P = 0.096) (Figure 7). Subgroup analysis revealed that the high heterogeneity was mainly attributed to the studies by Arazi et al. (2017) and Cin et al. (2021), with an I^2^ value of 93.0%. Different testing methods may be a source of heterogeneity; furthermore, the varying demands for change-of-direction ability across different sports disciplines may also contribute to increased heterogeneity. However, sensitivity analysis demonstrated the robustness of the results, indicating that this pooling approach is insufficient to alter the conclusions. Publication bias tests (Table 3) demonstrated the robustness of the pooled results: Begg’s test (P = 0.221) and Egger’s test (P = 0.066).

## 4 Discussion

Specifically, this study’s results demonstrate that CS outperforms TS in enhancing sprinting ability, explosive power, and lower limb explosive power, with statistically significant overall improvements. Subgroup analyses reveal that CS showed greater pooled effects than TS in boosting maximum strength, explosive power, lower limb explosive power, and sprinting ability among volleyball players. Additionally, for soccer players, CS shows greater efficacy in improving peak power. These findings underscore CS’s distinct advantages in optimizing athletic performance across different sports, highlighting its potential as a superior training modality for enhancing athletes’ specific physical attributes.

Volleyball players derive the most significant training benefits from CS among the five categories of sports projects included in the literature. The improvements in their maximum strength, sprinting ability, and overall explosive power are significantly greater than those achieved with TS. In volleyball matches, athletes predominantly perform short-distance, rapid movements and single maximal-effort jumps, with relatively low demands for sustained fatigue resistance and deceleration maneuvers. As posited by Silva et al. (2016), such sport characteristics lead to the predominance of the phosphagen energy system during competition, accompanied by a higher engagement of type II muscle fibers. The objective of employing CS in training aligns with enhancing the proportion of phosphagen system energy supply, mitigating the fatigue of type II muscle fibers, and accelerating movement speed to elicit corresponding physiological adaptations. Additionally, the rest-to-work ratio of 3.5:1 (Giatsis et al., 2020) closely resembles the structure of cluster sets, further highlighting the unique value of CS in improving volleyball performance. Consequently, it can be inferred that CS may exhibit high transferability and applicability to sports with movement patterns similar to volleyball, such as sepaktakraw (Hidayat et al., 2020).

Although existing research has confirmed that CS can effectively enhance average/peak velocity during resistance training (Wetmore et al., 2019), its impact on power output remains controversial (Hansen et al., 2011; Hardee et al., 2012). Some studies have indicated no significant difference in power improvement between CS and TS, possibly due to similar intensities (especially loads) used between CS and TS in intervention experiments (Suchomel et al., 2016). However, this study’s meta-analysis of peak power output in soccer players revealed a significant advantage of CS. This could be closely related to the movement characteristics of soccer: players frequently perform sudden stops, accelerations, direction changes, passing, and shooting during slow-speed runs (Sarmento et al., 2014), actions that rely on fast-twitch muscle fiber recruitment to generate peak power. Notably, the two studies included in this research (Hansen et al., 2011; Zhu et al., 2024) used the Wingate cycle ergometer and BS test, respectively. The former simulates on-field acceleration, while the latter effectively replicates jumping for headers and defensive positioning, together explaining CS’s superiority. Additionally, While the present study pooled studies that measured peak power using the Wingate, CMJ, SJ, and BJ methods based on the research of Martinez et al. (2016) and Krishnan et al. (2017), it should be noted that different sports have specific requirements for peak power testing methods: soccer, with its large playing field allowing gradual acceleration to peak power, is better evaluated by the Wingate test, whereas volleyball and table tennis, under restrictions by smaller courts, prefer short-duration explosive tests like SJ and CVJ. The meta-analysis of explosive power, sprint ability, and agility in soccer players showed ES just crossing the minimal clinically important difference. The authors speculate that CS’s potential to improve soccer-specific abilities may not be fully realized due to limited study inclusion and small sample sizes. Future research is recommended to use larger samples and sport-specific testing methods for further validation. Furthermore, two studies included in this review (Rong and Xiu, 2024; Zhu et al., 2024) reported average power output results for volleyball and table tennis players post-intervention, indicating that while CS and TS showed no difference in enhancing average power output, both outperformed the control group, and plyometric training was more effective than resistance training.

Given that only one study was included for each of rugby, table tennis, judo, and badminton, the results available for meta-analysis were limited. Subgroup analyses revealed no statistically significant differences in performance improvements between CS and TS across these four sports. Specifically, according to the match characteristics of rugby (Duthie et al., 2003), physical ability requirements vary significantly among players in different positions: quarterbacks rely more on upper-body explosive power without the need for sustained physical confrontation like tight ends and fullbacks, while halfbacks and wide receivers prioritize speed and agility to elude defenders. This position specificity necessitates customized cluster structures, load prescriptions, and sport-specific testing methods when applying CS. In the contexts of table tennis (Kondrič et al., 2013) and judo (Schoof et al., 2024), the absence of rest intervals similar to those in CS (15–45s) (Haff et al., 2008) highlights a greater demand for endurance in sustained physical confrontation and maintaining high-quality movements or power output under fatigue. Existing research indicates that CS is equally effective as TS in improving muscular endurance, with no significant differences (Farias et al., 2019; Dias et al., 2020) or potential local specificity, such as greater improvements in local muscular endurance (e.g., core training) with cluster sets (Prestes et al., 2019). Badminton’s characteristic of “speed changes, sudden stops and starts” primarily relies on the phosphagen energy system (Manrique and González-Badillo, 2003). While extended rallies against high-level opponents increase the contribution of glycolytic energy, neither scenario includes CS-like rest intervals lasting several seconds. Consequently, badminton training requires a nuanced approach: CS may be more beneficial for addressing weaknesses in smash height or racket speed, whereas TS might be preferred to enhance the ability to maintain movement quality during prolonged rallies.

The intervention cycles included in this paper ranged from 4 to 12 weeks, categorizing them as long-term interventions. During the literature collection, several studies were found to explore the acute effects of CS on athletes across different sports. Although methodological differences precluded their inclusion in the meta-analysis, their findings hold significant guiding value for the practical application of CS. For instance, in terms of post-activation potentiation (PAP), Nickerson et al. (2018) administered CS with 30-s (CS30) and 60-s (CS60) repeated intervals to soccer players, observing that while CS60 enhanced barbell movement speed, CS30 elicited a more pronounced PAP effect. Kurt et al. (2018) investigated CS and TS in rugby athletes and found that insufficient sets, jump counts, and intensity failed to alter the stiffness of the muscle-tendon complex (MTC), thereby preventing PAP induction. They speculated that performing more than 20 jumps per training session and using box heights exceeding 30 cm are optimal for enhancing the Reactive Strength Index (RSI) and leg rigidity (Kieg).

Physiologically and biochemically, Girman et al. (2014) measured growth hormone (GH), cortisol (C), blood lactate (BL), CVJ, and SLJ in weightlifters before, immediately after, and 15–30 min post-training with CS and TS. The results showed that CS imposed a lighter metabolic load and better sustained jumping ability. Arazi et al. (2016) analyzed 8-hydroxy-2′-deoxyguanosine (8-OHdG), 4-hydroxy-2-nonenal (4-HNE), and uric acid in volleyball players after CS and TS, revealing that resistance training induced DNA damage and oxidative stress, which were more severe under TS, indicating higher metabolic stress with TS. Furthermore, a study by Almeida et al. (2024) demonstrated that lactate concentrations were lower following CS. This finding implies that CS may not be an optimal training approach for enhancing performance in sports events such as the 400 m and 800 m races, which predominantly rely on fast glycolytic energy systems, and the study also reported that the Rating of Perceived Exertion (RPE) was significantly higher during CS sessions. This elevated RPE suggests that athletes may subjectively perceive greater fatigue when engaged in CS, potentially affecting training adherence and recovery dynamics.

In terms of movement quality and technique maintenance, higher training precision can improve training efficacy and reduce injury risks. For example, CS enhanced the maximum strength of specialized movements in gymnasts, improving competition stability (Schrer et al., 2019). Hardee et al. (2013) compared barbell trajectories during clean training with CS and TS, finding that cluster structure with rest intervals >20 s better preserved weightlifting technique than traditional sets. This highlights the potential advantages of CS in judged sports such as diving, gymnastics, and figure skating.

### 4.1 Practical applications

Common grouping patterns include “Intra-set Rest, Inter-repetition Rest, Rest Redistribution, Rest-Pause Method, and Equal Work-to-Rest Ratio” (Tufano et al., 2017). Brief rest intervals between training sets typically range from 15 to 45 s (Haff et al., 2008), with the exact duration tailored to the specific demands of individual sports.

In practice, any training structure must align with the unique requirements of the target sport, among which intermission timing and set configuration are particularly critical. Findings from this study indicate the following:

1. CS exhibits a high degree of compatibility with the competitive characteristics of intermittent high-explosive sports (e.g., volleyball, soccer), yielding significant improvements in training efficacy.
2. In contrast, TS may be more suitable for sports requiring sustained fatigue resistance (e.g., judo), as it facilitates prolonged muscle tension duration.

Furthermore, CS training demonstrates distinct advantages in periodized training programs:

1. During the off-season preparation phase, CS enables athletes to complete more repetitions and accumulate greater total strength training volume under equivalent load conditions, thereby promoting skeletal muscle hypertrophy more effectively.
2. In the mid-season, when athletes face competition-induced fatigue while needing to maintain training intensity and reduce overall load—rational application of CS can effectively mitigate declines in movement speed and power within controlled total load parameters, enabling higher-intensity training. This approach not only ensures that training intensity meets or exceeds competitive requirements but also prevents fatigue accumulation associated with overtraining, thereby reducing the risk of fatigue-related training injuries.
3. Additionally, in the pre-competition phase, CS can be utilized to elicit post-activation potentiation (PAP), enhancing athletic performance without inducing excessive fatigue.

Overall, reducing fatigue is recognized as a key factor in optimizing movement quality (Hardee et al., 2013)—a benefit that is particularly pronounced in multi-joint sports with high and complex skill demands, where CS training demonstrates more substantial advantages. Theoretically, CS contributes positively to speed development, which directly influences power output. Given that improving movement speed and power constitutes a primary objective in most sports (with rare exceptions), CS holds broad applicability across numerous athletic disciplines.

However, the extended interval times inherent to CS may increase the time cost of group training, presenting a challenge for populations with limited training time (e.g., collegiate athletes). This constraint, however, can potentially be addressed through the adoption of rest redistribution strategies.

### 4.2 Strengths and limitations

This study combined 11 research works to compare the effects of CS and TS on multiple indices, and conducted subgroup analyses based on athletes’ specific sports. The results demonstrated the potential advantages and disadvantages of CS compared with TS, indicating that volleyball players are more suitable for applying CS in training than athletes in soccer, rugby, table tennis, judo, and badminton. However, the application of CS in soccer shows untested potential that requires further verification.

Nonetheless, the reliability of these conclusions is somewhat limited due to: (1) the limited number of included studies and small sample sizes; (2) the fact that only a few articles reported on the same outcome measures this also limits the possibility of conducting subgroup analyses for different studies; (3) the absence of cross-comparative analyses in existing research regarding index differences among athletes from different sports after receiving the same intervention; (4) The variability in athletes’ competitive levels may have had some impact on the results. Future studies could expand sample sizes, recruit athletes with higher competitive levels from diverse sports, conduct multidimensional tests within the same study to compare the effects of CS and TS on different athletic qualities, and deeply explore the specific differences in applying CS across various sports.

## 5 Conclusion

CS demonstrates superior effects in enhancing sprint ability, explosive power, and lower-limb explosiveness compared to TS. It shows comparable efficacy to TS in improving peak power, agility, and maximum strength. Specifically, CS significantly outperforms TS in reinforcing maximum strength, explosive power, and sprint capacity among volleyball athletes. For soccer players, CS exhibits better effects than TS in enhancing peak power.

Acute responses induced by CS can elicit PAP while reducing physiological and psychological fatigue. It not only decreases metabolic stress and blood lactate levels but also effectively maintains the motor quality of movement techniques. Based on these findings, it is recommended to scientifically and rationally select and apply CS methods according to the training objectives and requirements of different sports.

## Data Availability

The original contributions presented in the study are included in the article/Supplementary Material, further inquiries can be directed to the corresponding authors.
