# Supplementary material for: The specificity of cluster training effects in sports: a systematic review and meta-analysis
Source: Front Physiol. 2026 Jan 19;16:1722401. doi: 10.3389/fphys.2025.1722401 (PMC12862356; doi:10.3389/fphys.2025.1722401)
Supplement: Supplementary file 1 [file Table1.DOCX]

**Table 1. Characteristics of included studies.**

| Study | Sport | Group | Age(years) | Height(cm) | Body weight(kg) | Sex(man/female) | Intervention characteristics | | | | | | | | Outcome measure |
| --- | --- | --- | --- | --- | --- | --- | --- | --- | --- | --- | --- | --- | --- | --- | --- |
|  |  |  |  |  |  |  | Frequency (times/week) | Stage(week) | Training content | Intensity(%1RM) | Set | Repetition | Rest between set(S) | CS rest interval (S) |  |
| Arazi et al. 2018 (2) | Volleyball | CS | 18.2±2.4 | 161±6 | 54.5±6.6 | 0/10 | 3 | 1-4 | BS, BP, DL, MP | 60-80 | 3 | 1×10, 2×5, 1×4+1×6 | 90–120 | 20-30 | 1RM (BS, BP, MP, DL), CVJ, peak power output (CVJ), 4×9mshuttle run, 20m sprint |
|  |  |  |  |  |  |  |  | 5-8 | SJ, EBP, DL, PC | 30-90 |  | 1×6, 2×3, 3×5×2, 3×3×2, 3×1×2 | 120–180 | 10-30 |  |
|  |  | TS | 18.7±1.5 | 166±5 | 56.5±9.0 | 0/10 |  | 1-4 | BS, BP, DL, MP | 60-80 |  | 8/10 | 120 | — |  |
|  |  |  |  |  |  |  |  | 5-8 | SJ, EBP, DL, PC | 30-90 |  | 4/6/8, 2×(1×6), 2×(1×4), 2×(1×3) | 180 | — |  |
| Merve et al.2021 (7) | Volleyball | CS | 27.1±1.60 | 195.7±1.49 | 94.14±1.65 | 14/0 | 3 | 1-6 | BP, MP, BS, DL, PO | 85 | 3 | 2×3 | 80 | 20 | 1RM (BS, BP, PO, DL) CVJ, T test, 10-20m sprint |
|  |  | TS | 27.4±1.37 | 194.4±1.89 | 93.6±1.25 | 14/0 |  |  |  |  |  | 6 | 120 | — |  |
| Rong et al.2024 (38) | Volleyball | CS-COX | 18.3±0.4 | 182.6±7.5 | 78.5±6.2 | 8/0 | 2 | 1-6 | BS, LP, SJ, LJ | 65 | 4 | 2×5/6/7/8 | 60 | 30 | 1RM (LP, BS) peak power output (Wingate) |
|  |  | TS-COX | 18.5±0.4 | 183.1±6.4 | 77.4±5.1 | 8/0 |  |  |  |  |  | 10/12/14/16 | 90 | — |  |
| Ersöz et al.2022 (12) | Soccer | AEL-CS | 18.78±0.83 | 166.73±8.61 | 69.59±6.03 | 16/0 | 2 | 1-4 | BS, HP | Eccentric 80%/Concentric 50% | 3 | 2×4 | 120 | 20 | 1RM (HP, BS), 10-20-30m sprint, Illinois test |
|  |  | AEL-TS |  |  |  | 16/0 |  |  |  |  |  | 8 | 180 | — |  |
| Öztürk et al.2025 (35) | Soccer | CS | 18.12±0.35 | 174±2 | 73.50 ± 3.62 | 8/0 |  | 1-8 | CVJ, BJ, TJ, HJ, DJ | Body weight | 8-10 | 2-3 | — | 10 | CVJ, 10-20-30m sprint, Zigzag test |
|  |  | TS | 18.50±0.53 | 175±4 | 72.25 ± 3.32 | 8/0 |  |  |  |  | 2-3 | 8-10 | 70/90 | — |  |
| Yilmaz et al. 2021 (51) | Soccer | CS | 14.88±0.92 | 173.2±8.9 | 57.38±7.79 | 9/0 | 2 | 1 | SLJ, SJ, AF, DLH | Body weight | 10 | 1×2 | — | 10 | CVJ, SJ, SLJ, 10m-20m sprint, Zigzag test |
|  |  |  |  |  |  |  |  | 2-3 | SLJE, SJ, DLH-LCH-FCH |  |  | 1×2 | — | 10 |  |
|  |  |  |  |  |  |  |  | 4-5 | SLJ, SJ, AF, DLH, LCH, SSJ |  |  | 1×2 | — | 10 |  |
|  |  |  |  |  |  |  |  | 6 | SLJ, SJ, DLH, LCH, FCH |  |  | 1×2 | — | 10 |  |
|  |  | TS | 15.25±0.70 | 171.2±5.9 | 54.01±8.33 | 8/0 |  | 1 | SLJ, SJ, AF, DLH |  | 2 | 10 | 90 | — |  |
|  |  |  |  |  |  |  |  | 2-3 | SLJE, SJ, DLH-LCH-FCH |  |  |  |  |  |  |
|  |  |  |  |  |  |  |  | 4-5 | SLJ, SJ, AF, DLH, LCH, SSJ |  |  |  |  |  |  |
|  |  |  |  |  |  |  |  | 6 | SLJ, SJ, DLH, LCH, FCH |  |  |  |  |  |  |
| Zarezadeh et al.2013 (53) | Soccer | CS | 24.68±3.13 | 176±0.41 | 71.68±6.85 | 11/0 | 3/4 | 1-4 | BS, LE, LC, HR, FC, BC, BP, IBP, LPD, BTNPWB, OPWB | 60-80 | 3 | 9 | 60-90 | — | 1RM BS, peak power output (BS) |
|  |  |  |  |  |  |  |  | 5-7 | BS, LG, LC, FC, BP | 85 | 3 | 1×5 | 120 | 10-30 |  |
|  |  |  |  |  |  |  |  | 8-10 | BS, SJ, BPT | 30-80 | 5 | 1×5 | 120 | 10-30 |  |
|  |  |  |  |  |  | 11/0 |  | 1-4 | BS, LE, LC, HR, FC, BC, BP, IBP, LPD, BTNPWB, OPWB | 60-80 | 3 | 9 | 60-90 | — |  |
|  |  | TS |  |  |  |  |  | 5-7 | BS, LG, LC, FC, BP | 85 | 3 | 5 | 180 | — |  |
|  |  |  |  |  |  |  |  | 8-10 | BS, SJ, BPT | 30-80 | 5 | 5 | 180 | — |  |
| Hansen et al. 2011 (18) | Rugby | CS | 27.8±4.5 | 185±10 | 99.7±10.5 | 9/0 | 2 | 1-2 | FS, CP | 80-90 | 5 | 1×6, 2×3 | 120-180 | 0-30 | 1RM BS, peak power output (0, 20, 40, 60kg SJ) |
|  |  |  |  |  |  |  |  | 3-4 | BS, CP | 80-95 |  | 1×5, 1×2+1×3 | 120-180 | 0-30 |  |
|  |  |  |  |  |  |  |  | 5-6 | BOXS, PC | 20-95 |  | 1×6, 3×1 | 120-180 | 0-10 |  |
|  |  |  |  |  |  |  |  | 7-8 | BS, SJ, PC |  |  | 1×3/4/5, 2×2, 3×1 | 120-180 | 0-20 |  |
|  |  | TS | 25.7±4.5 | 193±10 | 107.3±6.7 | 9/0 |  | 1-2 | FS, CP | 80-90 |  | 4/6/8 | 180 | — |  |
|  |  |  |  |  |  |  |  | 3-4 | BS, CP | 80-95 |  | 3/5/7 | 180 | — |  |
|  |  |  |  |  |  |  |  | 5-6 | BOXS, PC | 20-95 |  | 3/4/5/6 | 180 | — |  |
|  |  |  |  |  |  |  |  | 7-8 | BS, SJ, PC |  |  | 3/4/5 | 180 | — |  |
| Zhu et al. 2024 (54) | Table tennis | CS-PT | 19.4±1.2 | 179.4±4.1 | 77.8±5.3 | 8/0 | 3 | 1-8 | MBCP, MBS, STC, LMBD | 5% Body weight | 3/4 | 2×5/6 | 60 | 30 | MBT, 1RM BP, peak power output (Wingate) |
|  |  | TS-PT | 19.7±1.4 | 180.8±5.5 | 79.2±5.8 | 8/0 |  |  |  |  |  | 10/12 | 90 |  |  |
|  |  | CS-RT | 19.5±1.8 | 179.4±4.6 | 78.8±4.2 | 8/0 |  |  | BP, SP, DFR, CPS |  |  | 2×5/6 | 60 | 30 |  |
|  |  | TS-RT | 19.6±1.1 | 178.8±4.5 | 76.2±4.8 | 8/0 |  |  |  |  |  | 10/12 | 90 |  |  |
| Harris et al. 2024 (21) | Judo | CS | 17.1±0.8 | 168.6±8.3 | 61.1±9.0 | 6/5 | 3 | 1-4 | PC, BOXS, DL, SS, SLRD, PJ, PBR, BP, SABOR, LPP, WPU | 65-85 | 4 | 2×3/5 | — | — | CVJ, 1RM (BS, BP) |
|  |  |  |  |  |  |  |  | 5-8 | BS, DL, SS, SLRD, IBP, PBR, SSP, WPU | 80-85 | 3/4 | 2×3/4 | — | 45 |  |
|  |  |  |  |  |  |  |  | 8-12 | MS, HT, BAT, BT, JS, HHP, BS, CP |  | 3 | 2×3 | — | 45 |  |
|  |  | TS | 17.8±1.4 | 171.8±5.9 | 65.6±6.6 | 7/4 |  | 1-4 | PC, BOXS, DL, SS, SLRD, PJ, PBR, BP, SABOR, LPP, WPU | 65-85 | 4 | 6/10 | — | — |  |
|  |  |  |  |  |  |  |  | 5-8 | BS, DL, SS, SLRD, IBP, PBR, SSP, WPU | 80-85 | 3/4 | 5-6/8 | — | — |  |
|  |  |  |  |  |  |  |  | 8-12 | MS, HT, BAT, BT, JS, HHP, BS, CP |  | 3 | 6 | — | — |  |
| Chen et al. 2023 (6) | Badminton | CS | 15.13±1.356 | 171.63±5.290 | 60.50±7.764 | 8/0 | 2 | 1-6 | BS | 80 | 4 | 2×3 | 90 | 30 | 1RM BS, 30m sprint, CVJ |
|  |  | TS | 15.25±1.282 | 173.38±9.257 | 60.88±10.494 | 8/0 |  |  |  |  |  | 6 | 120 | — |  |

**Abbreviations: AEL: accentuated eccentric load; AF: angle flip; BAT: bridge and toss (dead ball) (osaekomi-waza); BC: barbell curl; BJ: broad jump; BP: bench press; BPT: bench press throw; BOXS: box squat; BS: back squat; BT: bench throws (smith machine); BTNPWB: behind the neck press; COX: resistance and plyometric training (complex); CP: clean pull; CVJ: countermovement vertical jump；DFR: dumbbell front rise; DL: deadlift; DLH: double leg hop; DJ: drop jump; EBP: explosive bench press; FC: french curl; FCH: front cone hop; FS: front squat; HHP: hang high pull; HJ: hurdle jump; HT: hip thrust; HR: heel raise; IBP: Incline bench press; JS: Jump shrug; LC: leg curl; LCH: lateral cone hop; LE: leg extension; LG: lunge; LJ: lunge jump; LMBD: lying medicine ball drop; LP: leg press; LPD: lat pull down; LPP: landmine push press; MBCP: medicine ball chest pass; MBS: medicine ball slams; MBT:medicine ball throw MS: midthigh snatch; PT: plyometric training; RT: resistance training; MP: military press; OPWB: overhead press with barbell; PBR: Prone bench (seal) row; PC: power clean; PJ: push jerk; PO: pull over; SJ: squat jump; SLJ: standing long jump; SSJ: split squat jump; SABOR: single-arm bent-over row; STC: seated throw circuit; SP: shoulder press; CPS: cable pulldowns; SS: split squat; SSP: seated shoulder press; TJ: tuck jump; WPU: weighted pull-up.**

**Table 2 Quality evaluation results of included literature**

| **Study**  **Criterion** | Arazi et al.,2018 | Merve et al.,2021 | Rong et al.,2024 | Ersöz et al.,2022 | Öztürk et al.,2025 | Yilmaz et al.,2021 | Zarezadeh et al.,2013 | Hansen et al.,2011 | Zhu et al.,2024 | Harris et al.,2024 | Chen et al.,2023 |
| --- | --- | --- | --- | --- | --- | --- | --- | --- | --- | --- | --- |
| **Eligibility criteria specified(1point)** | 1 | 1 | 1 | 1 | 1 | 1 | 1 | 1 | 1 | 1 | 1 |
| **Randomization specified(1point)** | 0 | 0 | 0 | 0 | 0 | 0 | 0 | 0 | 0 | 0 | 1 |
| **Allocation concealment(1point)** | 0 | 1 | 0 | 1 | 0 | 0 | 0 | 1 | 0 | 0 | 1 |
| **Groups similar at baseline(1point)** | 1 | 1 | 1 | 1 | 1 | 1 | 1 | 1 | 1 | 1 | 1 |
| **Blinding of assessor(1point)** | 1 | 1 | 1 | 1 | 1 | 1 | 1 | 1 | 1 | 1 | 1 |
| **Outcome measures assessed in 85% of patients(3point)** | 2 | 1 | 2 | 1 | 2 | 1 | 1 | 1 | 2 | 1 | 1 |
| **Intention-to-treat analysis(1point)** | 0 | 0 | 0 | 0 | 0 | 0 | 0 | 0 | 0 | 0 | 0 |
| **Between-group statistical comparisons reported(2point)** | 2 | 2 | 2 | 2 | 2 | 2 | 2 | 2 | 2 | 2 | 2 |
| **Point measures and measures of variability for all reported outcome measures(1point)** | 1 | 1 | 1 | 1 | 1 | 1 | 1 | 1 | 1 | 1 | 1 |
| **Activity monitoring in control groups(1point)** | 1 | 1 | 1 | 1 | 1 | 1 | 1 | 1 | 1 | 1 | 0 |
| **Relative exercise intensity remained constant(1point)** | 1 | 0 | 1 | 1 | 1 | 1 | 0 | 1 | 1 | 1 | 0 |
| **Exercise volume and energy expenditure(1point)** | 1 | 1 | 1 | 1 | 1 | 1 | 1 | 1 | 1 | 1 | 1 |
| Total points | 11 | 10 | 11 | 11 | 12 | 10 | 9 | 11 | 11 | 10 | 11 |
| Quality | Good | Good | Good | Good | Excellent | Good | Good | Good | Good | Good | Good |

**Table 3. Results of Egger's Test and Begg's Publication Bias Test**

| Outcome measure | Begg's test | Egger's test |
| --- | --- | --- |
| Maximal force | 0.552 | 0.116 |
| Upper-body maximal strength | 0.293 | 0.010 |
| Lower-body maximal strength | 0.732 | 0.302 |
| Explosive power | 0.032 | 0.214 |
| Lower-body explosive strength | 0.174 | 0.238 |
| Peak power output | 0.754 | 0.735 |
| Sprinting ability | 0.837 | 0.946 |
| Agility and speed | 0.221 | 0.066 |
