# Supplementary material for: The specificity of cluster training effects in sports: a systematic review and meta-analysis
Source: Front Physiol. 2026 Jan 19;16:1722401. doi: 10.3389/fphys.2025.1722401 (PMC12862356; doi:10.3389/fphys.2025.1722401)
Supplement: Supplementary file 2 [file Table2.DOCX]

Embase:

#1: 'resistance training'/exp

#2: 'resistance exercise'/exp OR 'resistance exercise' OR (('resistance'/exp OR resistance) AND ('exercise'/exp OR exercise)) OR 'training, resistance':ab,ti OR 'strength training':ab,ti OR 'weight-lifting strengthening program':ab,ti OR 'weight-lifting exercise program':ab,ti OR 'weight-bearing strengthening program':ab,ti OR 'weight-bearing exercise program':ab,ti

#3: #1 OR #2

#4: 'cluster set' OR (('cluster'/exp OR cluster) AND set) OR 'cluster training':ab,ti OR cluster:ab,ti OR 'cluster loading':ab,ti OR 'cluster type':ab,ti OR 'rest pause':ab,ti OR 'traditional set':ab,ti OR 'intra set':ab,ti OR 'inter rep':ab,ti OR 'work-to-rest ratio':ab,ti OR 'rest redistribution':ab,ti OR 'rest loading':ab,ti

#5: 'athlete'/exp

#6: 'athlete'/exp OR athlete OR 'athlete, professional':ab,ti OR 'athletes, professional':ab,ti OR 'elite athletes':ab,ti OR 'athlete, elite':ab,ti OR 'college athletes':ab,ti OR 'athlete, college':ab,ti OR sportswoman:ab,ti OR sportsman:ab,ti OR player:ab,ti OR players:ab,ti OR 'professional athletes':ab,ti

#7: #5 OR #6

#8: #3 AND #5 AND #7

Web of science:

#1: ((((((TS=(resistance exercise)) OR TS=(Training, Resistance)) OR TS=(Strength Training)) OR TS=(Weight-Lifting Strengthening Program)) OR TS=(Weight-Lifting Exercise Program)) OR TS=(Weight-Bearing Strengthening Program)) OR TS=(Weight-Bearing Exercise Program) and Preprint Citation Index

#2: (((((((((((TS=(cluster set)) OR TS=(cluster training)) OR TS=(cluster)) OR TS=(cluster loading)) OR TS=(cluster-type)) OR TS=(rest-pause)) OR TS=(traditional set)) OR TS=(intra set)) OR TS=(inter rep)) OR TS=(work-to-rest ratio)) OR TS=(rest redistribution)) OR TS=(rest-loading) and Preprint Citation Index

#3: (((((((((((TS=(Athlete)) OR TS=(Professional Athletes)) OR TS=(Athlete, Professional)) OR TS=(Athletes, Professional)) OR TS=(Elite Athletes)) OR TS=(Athlete, Elite)) OR TS=(College Athletes)) OR TS=(Athlete, College)) OR TS=(sportswoman)) OR TS=(sportsman)) OR TS=(player)) OR TS=(players) and Preprint Citation Index

#4: #3 AND #2 AND #1 and Preprint Citation Index

#5: #1 AND #2 AND #3 and Preprint Citation Index

#6: #1 AND #2 AND #3 and Preprint Citation Index

Pubmed:

**(((((((((resistance exercise) OR (Training, Resistance)) OR (Strength Training)) OR (Weight-Lifting Strengthening Program)) OR (Weight-Lifting Exercise Program)) OR (Weight-Bearing Strengthening Program)) OR (Weight-Bearing Exercise Program))) AND ((((((((((((cluster set)) OR (cluster training) OR (cluster)) OR (cluster loading)) OR (cluster-type)) OR (rest-pause)) OR (traditional set)) OR (intra set)) OR (inter rep)) OR (work-to-rest ratio)) OR (rest redistribution)) OR (rest-loading))) AND ((((((((((((Athlete) OR (Professional Athletes)) OR (Athlete, Professional)) OR (Athletes, Professional)) OR (Elite Athletes)) OR (Athlete, Elite)) OR (College Athletes)) OR (Athlete, College)) OR (sportswoman)) OR (sportsman)) OR (player)) OR (players))**

Cochrane:

**(resistance exercise) OR (Training, Resistance)) OR (Strength Training) OR (Weight-Lifting Strengthening Program) OR (Weight-Lifting Exercise Program) OR (Weight-Bearing Strengthening Program)) OR (Weight-Bearing Exercise Program) AND (cluster set) OR (cluster training) OR (cluster) OR (cluster loading) OR (cluster-type) OR (rest-pause) OR (traditional set) OR (intra set) OR (inter rep) OR (work-to-rest ratio) OR (rest redistribution) OR (rest-loading) AND (Athlete) OR (Professional Athletes) OR (Athlete, Professional) OR (Athletes, Professional) OR (Elite Athletes)) OR (Athlete, Elite) OR (College Athletes) OR (Athlete, College) OR (sportswoman) OR (sportsman) OR (player) OR (players)**
