# Supplementary material for: The specificity of cluster training effects in sports: a systematic review and meta-analysis
Source: Front Physiol. 2026 Jan 19;16:1722401. doi: 10.3389/fphys.2025.1722401 (PMC12862356; doi:10.3389/fphys.2025.1722401)
Supplement: Supplementary file 3 [file Table3.DOCX]

| **Maximal strength** | Sports | CS | | | TS | | |
| --- | --- | --- | --- | --- | --- | --- | --- |
| Study |  | n1 | mean1 | sd1 | n2 | mean2 | sd2 |
| Arazi et al.,2018（BS） | 1 | 10 | 3.6 | 0.652 | 10 | 3.3 | 1.587 |
| Merve et al.,2021（BS） | 1 | 14 | 8.7 | 9.834434402 | 14 | 3.9 | 6.950194242 |
| Rong et al.,2024（BS） | 2 | 8 | 13.018 | 16.00931507 | 8 | 12.426 | 11.68981099 |
| Ersöz et al.,2022（BS） | 2 | 16 | 15.5 | 15.35411346 | 16 | 18.38 | 16.12167485 |
| Hansen et al.,2011（BS） | 3 | 9 | 25.3 | 25.15134191 | 9 | 36.9 | 22.03542602 |
| Harris et al.,2024（BS） | 5 | 11 | 1.927 | 23.0813992 | 11 | 3.579 | 18.77481337 |
| Zarezadeh et al.,2013（BS） | 2 | 11 | 16.4 | 17.77006753 | 11 | 35.31 | 22.05357341 |
| Chen et al.,2023（BS） | 6 | 8 | 12.1 | 9.93021455 | 8 | 19.06 | 16.87909998 |
| Arazi et al.,2018（DL） | 1 | 10 | 2 | 0.6 | 10 | 2 | 0.7 |
| Merve et al.,2021（DL） | 1 | 14 | 4.8 | 8.954591001 | 14 | 2 | 7.303649773 |
| Ersöz et al.,2022（HT） | 2 | 16 | 15.31 | 15.12253947 | 16 | 16.69 | 14.40219775 |
| Rong et al.,2024（LP） | 2 | 8 | 21.577 | 27.77176473 | 8 | 21.075 | 25.81727342 |
| Merve et al.,2021（PO） | 1 | 14 | 5.1 | 6.945941261 | 14 | 2.7 | 5.252313776 |
| Arazi et al.,2018（MP） | 1 | 10 | 0.9 | 0.458257569 | 10 | 0.9 | 0.360555128 |
| Arazi et al.,2018（BP） | 1 | 10 | 2.1 | 0.888819442 | 10 | 2.1 | 0.793725393 |
| Merve et al.,2021（BP） | 1 | 14 | 5.3 | 7.24877231 | 14 | 2.3 | 6.82116559 |
| Harris et al.,2024（BP） | 5 | 11 | 6.8247 | 19.3624568 | 11 | 1.8958 | 25.05814475 |
| Zhu et al.2024(CS/TS-PT)（BP） | 4 | 8 | 4.6 | 11.00545319 | 8 | 4.5 | 9.801530493 |
| Zhu et al.2024(CS/TS-RT)（BP） | 4 | 8 | 7.6 | 11.75542428 | 8 | 7.1 | 10.81295519 |

| Sprint | Sports | CS | | | TS | | |
| --- | --- | --- | --- | --- | --- | --- | --- |
| Study |  | n1 | mean1 | sd1 | n2 | mean2 | sd2 |
| Merve et al.,2021（10） | 1 | 14 | -0.06 | 0.11 | 14 | -0.02 | 0.07 |
| Ersöz et al.,2022（10） | 2 | 16 | -0.07 | 0.07649183 | 16 | -0.03 | 0.10516178 |
| Öztürk et al.2025（10） | 2 | 8 | -0.14 | 0.07 | 8 | -0.16 | 0.060827625 |
| Yilmaz et al.2021（10） | 2 | 9 | -0.06 | 0.125299641 | 8 | -0.09 | 0.135277493 |
| Merve et al.,2021（20） | 1 | 14 | -0.2 | 0.122882057 | 14 | -0.05 | 0.105356538 |
| Arazi et al.,2018（20） | 1 | 10 | -0.03 | 0.8 | 10 | -0.01 | 0.31 |
| Ersöz et al.,2022（20） | 2 | 16 | -0.09 | 0.124430704 | 16 | -0.03 | 0.913540366 |
| Öztürk et al.2025（20） | 2 | 8 | -0.14 | 0.081853528 | 8 | -0.11 | 0.06244998 |
| Yilmaz et al.2021（20） | 2 | 9 | -0.09 | 0.195192213 | 8 | -0.06 | 0.230651252 |
| Ersöz et al.,2022（30） | 2 | 16 | -0.18 | 0.226099536 | 16 | -0.11 | 0.192779667 |
| Öztürk et al.2025（30） | 2 | 8 | -0.14 | 0.052915026 | 8 | -0.12 | 0.036055513 |
| Chen et al.,2023（30） | 6 | 8 | -0.14 | 0.233263799 | 8 | -0.07 | 0.245786493 |

| Explosive power | Sports | CS | | | TS | | |
| --- | --- | --- | --- | --- | --- | --- | --- |
| Study |  | n1 | mean1 | sd1 | n2 | mean2 | sd2 |
| Arazi et al.,2018（CVJ） | 1 | 10 | 2.4138 | 5.8621 | 10 | 1.5514 | 4.762509406 |
| Merve et al.,2021（CVJ） | 1 | 14 | 4.1 | 1.690354992 | 14 | 1.41 | 1.188991169 |
| Ersöz et al.,2022（CVJ） | 2 | 16 | 3.19 | 2.017448884 | 16 | 1.92 | 2.643633863 |
| Harris et al.,2024（CVJ） | 5 | 11 | 0.4587 | 16.62968672 | 11 | 0.2293 | 14.80554737 |
| Öztürk et al.2025（CVJ） | 2 | 8 | 5.83 | 0.650922422 | 8 | 5.19 | 1.254073363 |
| Chen et al.,2023（CVJ） | 6 | 8 | 4.85 | 5.693297199 | 8 | 2.51 | 5.749049226 |
| Yilmaz et al.2021（SJ） | 2 | 9 | 3.23 | 5.118564252 | 8 | 3.09 | 4.77799121 |
| Yilmaz et al.2021（SLJ） | 2 | 9 | 12 | 18.734994 | 8 | 11 | 23.2594067 |
| Zhu et al.2024(CS/TS-PT)（MB） | 4 | 8 | 60.5 | 46.45137242 | 8 | 45.5 | 42.30319137 |
| Zhu et al.2024(CS/TS-RT)（MB） | 4 | 8 | 40.3 | 47.48294431 | 8 | 29.1 | 43.25702255 |

| Agility and speed | Sports | CS | | | TS | | |
| --- | --- | --- | --- | --- | --- | --- | --- |
| Study |  | n1 | mean1 | sd1 | n2 | mean2 | sd2 |
| Öztürk et al.2025（zigzag） | 2 | 8 | -0.28 | 0.06244998 | 8 | -0.24 | 0.088881944 |
| Yilmaz et al.2021（zigzag） | 2 | 9 | -0.28 | 0.365923489 | 8 | -0.08 | 0.416173041 |
| Merve et al.,2021（T） | 1 | 14 | -1.03 | 0.392300905 | 14 | -0.23 | 0.243310501 |
| Ersöz et al.,2022（I） | 2 | 16 | -0.27 | 0.303856874 | 16 | -0.22 | 0.279565735 |
| Arazi et al.,2018（4×9） | 1 | 10 | -0.01 | 0.8 | 10 | -0.01 | 0.7 |

| Peak power output(w) | Sports | CS | | | TS | | |
| --- | --- | --- | --- | --- | --- | --- | --- |
| Study |  | n1 | mean1 | sd1 | n2 | mean2 | sd2 |
| Zhu et al.2024(CS/TS-PT)（wingate） | 4 | 8 | 59.5 | 30.84866286 | 8 | 59.8 | 34.11275421 |
| Zhu et al.2024(CS/TS-RT)（wingate） | 4 | 8 | 30.8 | 32.35104326 | 8 | 28 | 39.43006467 |
| Rong et al.,2024（wingate） | 2 | 8 | 102.894 | 60.00443661 | 8 | 67.524 | 56.27 |
| Arazi et al.,2018（CVJ） | 1 | 10 | 111.802 | 399.8402598 | 10 | 74.534 | 519.5544835 |
| Hansen et al.,2011（SJ 0kg） | 3 | 9 | 174.3 | 539.4339348 | 9 | 93 | 447.8018758 |
| Hansen et al.,2011（SJ 20kg） | 3 | 9 | 118.5 | 417.0544689 | 9 | 205.6 | 489.5622228 |
| Hansen et al.,2011（SJ 40kg） | 3 | 9 | 278.8 | 301.944018 | 9 | 21.6 | 488.3185538 |
| Hansen et al.,2011（SJ 40kg） | 3 | 9 | 161.5 | 298.0716021 | 9 | 106.2 | 561.132667 |
| Zarezadeh et al.,2013（BS） | 2 | 11 | 428.67 | 622.8593975 | 11 | 32.79 | 544.6105775 |

1: Volleyball, 2: Football, 3: Rugby, 4: Table tennis,5: Judo, 6: Badminton.

Stata code：

Import the original data: meta esize n1 mean1 sd1 n2 mean2 sd2, studylabel

Calculate the effect size: meta summarize, random(reml) subgroup

Draw the forest plot: meta forestplot

Draw the forest plot for the sensitivity analysis: meta forestplot, leaveoneout

Draw the Funnel Plotmeta funnelplot

Apply the Trim-and-Fill method to correct publication bias and draw the corrected funnel plot: meta trimfill, funnel
